# Two genetically diverse H7N7 avian influenza viruses isolated from migratory birds in central China

**DOI:** 10.1038/s41426-018-0064-7

**Published:** 2018-04-11

**Authors:** Haizhou Liu, Chaochao Xiong, Jing Chen, Guang Chen, Jun Zhang, Yong Li, Yanping Xiong, Runkun Wang, Ying Cao, Quanjiao Chen, Di Liu, Hanzhong Wang, Jianjun Chen

**Affiliations:** 1 CAS Key Laboratory of Special Pathogens and Biosafety, Wuhan Institute of Virology, Chinese Academy of Sciences, Wuhan, Hubei 430071 China; 2 Hubei Wildlife Rescue, Research and Development Center, Wuhan, Hubei 430074 China; 3 Center for Influenza Research and Early warning (CASCIRE), Chinese Academy of Sciences, Beijing, 100101 China; 4 University of Chinese Academy Sciences, Beijing, 101409 China

## Abstract

After the emergence of H7N9 avian influenza viruses (AIV) in early 2013 in China, active surveillance of AIVs in migratory birds was undertaken, and two H7N7 strains were subsequently recovered from the fresh droppings of migratory birds; the strains were from different hosts and sampling sites. Phylogenetic and sequence similarity network analyses indicated that several genes of the two H7N7 viruses were closely related to those in AIVs circulating in domestic poultry, although different gene segments were implicated in the two isolates. This strongly suggested that genes from viruses infecting migratory birds have been introduced into poultry-infecting strains. A Bayesian phylogenetic reconstruction of all eight segments implied that multiple reassortments have occurred in the evolution of these viruses, particularly during late 2011 and early 2014. Antigenic analysis using a hemagglutination inhibition test showed that the two H7N7 viruses were moderately cross-reactive with H7N9-specific anti-serum. The ability of the two H7N7 viruses to remain infectious under various pH and temperature conditions was evaluated, and the viruses persisted the longest at near-neutral pH and in cold temperatures. Animal infection experiments showed that the viruses were avirulent to mice and could not be recovered from any organs. Our results indicate that low pathogenic, divergent H7N7 viruses circulate within the East Asian-Australasian flyway. Virus dispersal between migratory birds and domestic poultry may increase the risk of the emergence of novel unprecedented strains.

## Introduction

The circulation of H7 subtype avian influenza viruses (AIV) in poultry has caused outbreaks in poultry and has even resulted in human infection for decades. Both the North American and Eurasian lineages of H7 viruses have been associated with human infection^1^, and the global distribution of this subtype has affected poultry in countries in Europe, America, Asia and Oceania^2^. To date, human infections have been caused by H7 subtypes of low pathogenic H7N2, H7N3, H7N7, and H7N9 and by highly pathogenic H7N3, H7N7, and H7N9^2, 3^. Before 2003, <20 sporadic cases of human infection with H7 viruses were reported in Europe and America^1^. In 2003, outbreaks of HPAI H7N7 struck poultry in several European countries, and 86 poultry workers and three of their family members were infected with this subtype in the Netherlands^4^. Among these infections, almost all infected persons developed mild to moderate conjunctivitis, and one person died from pneumonia and acute respiratory distress syndrome^4^. This outbreak represented the first H7 avian influenza outbreak in humans. H7N9 caused another H7 outbreak in 2013 in Eastern China^5, 6^. The virus responsible was a novel reassortant strain between wild bird-origin H7 and N9 viruses and poultry-infecting H9N2 viruses^7, 8^.

The majority of human H7 cases have been associated with exposure to domestic poultry, whereas the H7 viruses circulating in poultry are apparently linked to the subtype in wild birds. Therefore, surveillance of AIVs in wild birds, especially H7 subtype AIVs in migratory waterfowl, will contribute to a general understanding of this subtype in reservoir species and will benefit the identification of when and where the introduction of viruses from migratory birds to domestic poultry or mammals has occurred. Long-term surveillance of H7 influenza viruses in American wild aquatic birds and poultry indicates that four polyphyletic highly pathogenic H7N3 strains arose in poultry through the introduction of low pathogenic H7N3 viruses from the wild aquatic bird reservoir^9^. In Europe, H7N3 viruses isolated from wild ducks in Italy in 2001 appeared to be closely related at both the phenotypic and genetic levels to H7N3 strains that circulated in Italian turkeys in 2002–2003. In Eastern Asia, >5 years of surveillance of H7 viruses in wild birds and domestic ducks also noted such introduction events occurring from wild birds to poultry, with two H7 strains isolated from domestic ducks with the same gene constellations across all gene segments as viruses originating in wild birds^10^. These findings indicate that H7 viruses are repeatedly introduced from wild birds to poultry and might evolve into highly pathogenic strains.

In this study, we isolated two strains of H7N7 viruses from different wild aquatic birds and analyzed their genetic relationship with wild bird and domestic poultry viruses and antigenic differences between the two isolates and a 2013 H7N9 isolate, their pathogenic potential in mice and environmental stability under variable conditions of temperature and pH.

## Results

### Two divergent H7N7 strains recovered from different bird species in different wetlands

**Fig. 1:**
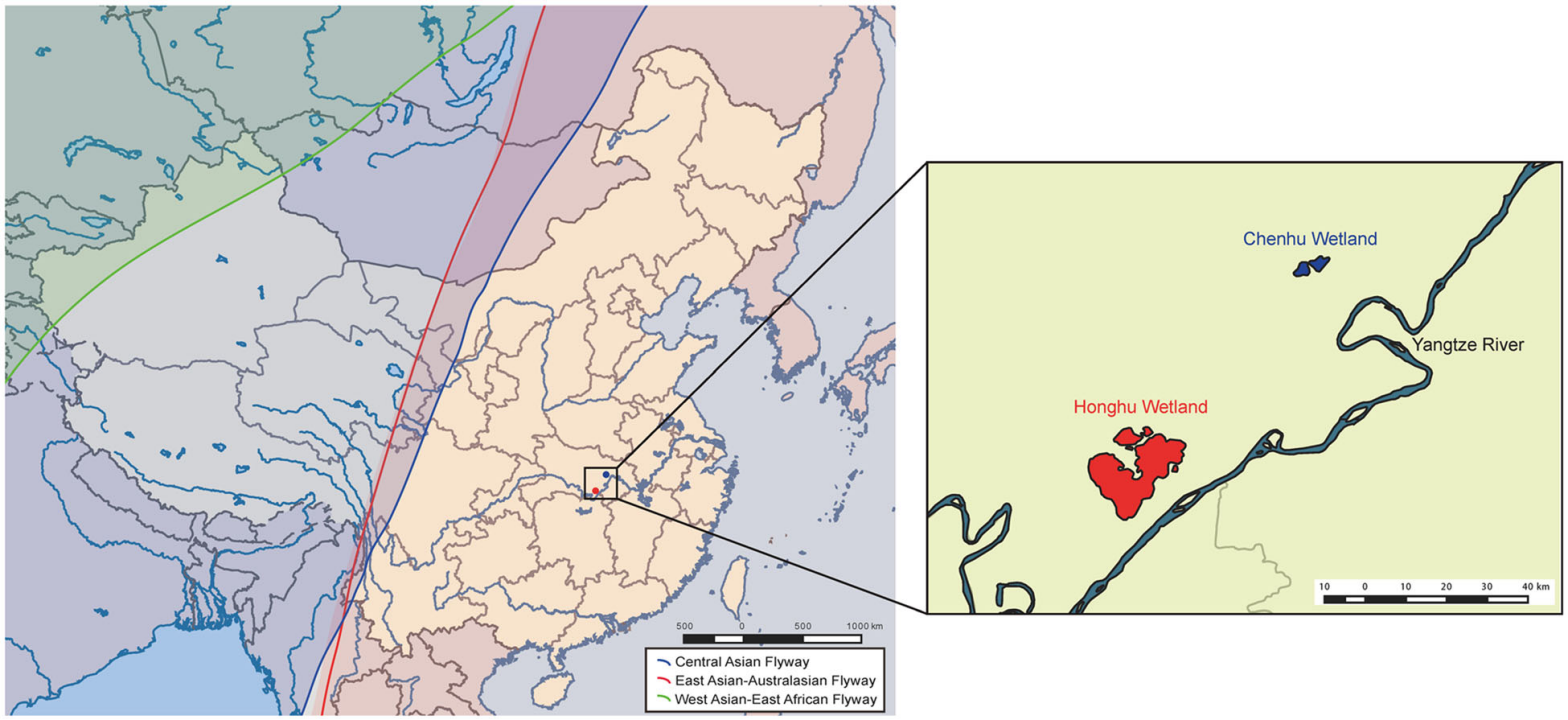
Map of migration flyways across China and sampling sites at which the H7N7 viruses were isolated. Both the Chenhu wetland and Honghu wetland are located in the East Asian-Australasian migratory flyway. The migration flyways in China were mapped by ArcGIS Desktop 10.2 software (http://www.esri.com/software/arcgis/arcgis-for-desktop/)

Active surveillance for AIVs in migratory waterfowl was undertaken in the Chenhu and Honghu wetlands—two major overwintering and stopover sites in Hubei Province that are located on the East Asian-Australasian flyway (Fig. 1). During December 2013–March 2014, a total of 1647 fresh droppings from migratory waterfowl were collected, and two H7N7 strains were recovered from different bird species and sampling sites. One strain was isolated from samples collected in Honghu wetlands in December 2013 and was designated A/Phalacrocorax carbo/Hubei/HH179/2013 (H7N7, HH179/H7N7). The other strain, A/Anser cygnoides/Hubei/CH1228/2014 (H7N7, CH1228/H7N7), was isolated from samples collected in Chenhu wetlands in February 2014 from a swan goose (*Anser cygnoides*) host. In addition, subtype H5N3 (*n* = 1), H5N6 (*n* = 3), H6N1 (*n* = 2), H6N2 (*n* = 2), and H6N8 (*n* = 1) were also isolated from fresh droppings, and the total AIVs positive rate was 0.65% (11 of 1647, data not shown).

The whole genome of the two H7N7 influenza viruses (HH179/H7N7 and CH1228/H7N7) were sequenced and compared with each other for genetic identity (Supplementary Table 1). Each gene segment from both viruses shared a relatively high sequence identity (96.2–98.9%), with the exception of the NA gene, which shared only 90.6%, indicating that the NA gene of the two H7N7 viruses was derived from divergent origins. When compared with human H7N7 isolates (A/Netherlands/219/2003 (H7N7) and A/Italy/3/2013 (H7N7)), the HA and NA gene of both H7N7 strains from waterfowl shared only 91.7–92% and 91.5–94.4% sequence identity with the human isolates (Supplementary Table 1), respectively, suggesting that the H7N7 virus from waterfowl had diverged from human H7N7 isolates. A BLAST search showed that several genes of HH179/H7N7 and CH1228/H7N7 were highly homologous (>99%) with various virus strains isolated from domestic poultry in China, indicating that gene flow occurs between wild birds and domestic poultry.

### Gene flow occurs between migratory waterfowl H7N7 viruses and domestic poultry strains

**Fig. 2:**
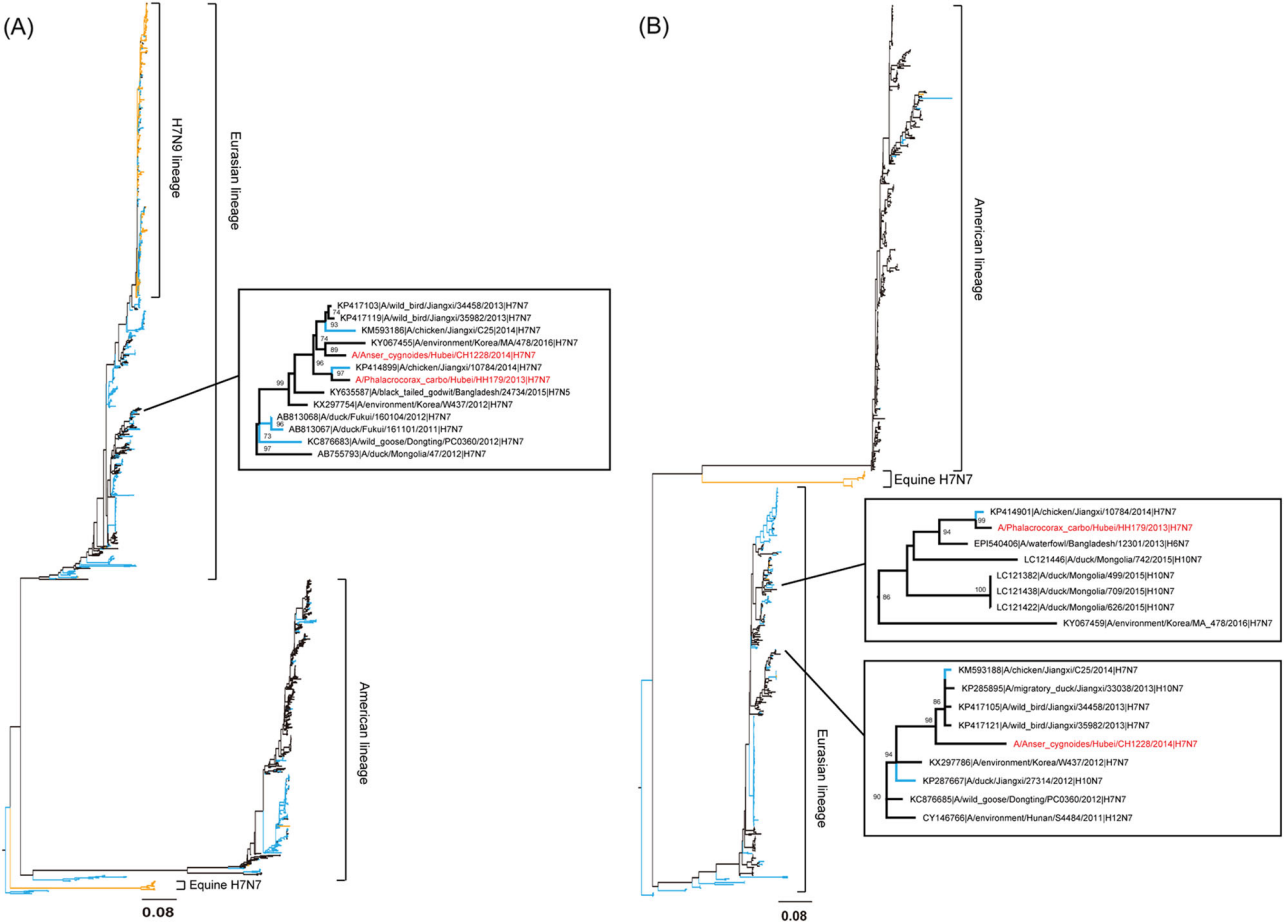
Maximum-likelihood phylogenetic trees for HA and NA segments of two H7N7 strains. **a** HA and **b** NA. Virus strain names are colored by host: blue for poultry, orange for human and mammals and black for wild birds and environment samples. The CH1228/H7N7 and HH179/H7N7 strain names are marked in red

Similar to BLASTN reports, the maximum-likelihood phylogenetic inferences by the RAxML program also suggested a highly divergent evolutionary picture of each gene (Fig. 2a, b and Supplementary Figure S1A-1F). Except for the HA segment, all seven remaining segments of these two influenza strains originated from different lineages and were interchanged among waterfowl, domestic poultry (chicken, duck, and goose) and wild birds. In the HA phylogenetic tree (Fig. 2a), H7 viruses clearly separated into Eurasian, American, and Equine lineages. Both HH179/H7N7 and CH1228/H7N7 fell into the Eurasian lineage and were clustered in a sub-branch that consisted of H7N7 viruses from wild birds and domestic poultry in central China and wild birds in other East Asian countries (Korea, Japan and Mongolia). In the NA tree (Fig. 2b), the N7 viruses showed a similar topology to the HA tree, with both HH179/H7N7 and CH1228/H7N7 falling into the Eurasian lineage but clustered in two separated sub-branches; in each sub-branch, both the H7N7 viruses clustered with other viruses from wild birds and domestic poultry in central China and East Asian countries. These results indicate that the HA and NA genes from both HH179/H7N7 and CH1228/H7N7 are highly similar to those found in viruses circulating in wild birds in the East Asian-Australasian flyway and that this gene pool has been introduced to domestic poultry in central China.

Phylogenetic tree analysis of the internal genes of our isolates showed that both HH179/H7N7 and CH1228/H7N7 were diversified and clustered into a separate sub-branch, although they all belong to the wild bird gene pool (Supplementary Figure S1A-1F). In the PB2 tree, CH1228/H7N7 was most closely related to H3Ny strains circulating in ducks in Southeast Asia, whereas the PB2 gene of HH179/H7N7 was highly similar to viruses in domestic poultry in central China (Supplementary Figure S1A). Both PB1 segments clustered with viruses from wild birds but differed in geographic location, with HH179/H7N7 and CH1228/H7N7 viruses clustering with strains from central China and Mongolia, respectively (Supplementary Figure S1B). In the PA tree, the HH179/H7N7 virus clustered with viruses from both wild birds and domestic poultry from Jiangxi province in central China, whereas CH1228/H7N7 strain was phylogenetically close to poultry H10N7 strains in Jiangxi province and H5N1 viruses in Bangladesh (Supplementary Figure S1C). Diversity between HH179/H7N7 and CH1228/H7N7 was also observed in the remaining NP, M, and NS genes. The NP segments of HH179/H7N7 and CH1228/H7N7 were closely related to a domestic poultry virus in Jiangxi province isolated in 2013, with HH179/H7N7 also demonstrating differences derived from wild bird viruses (Supplementary Figure S1D). In the M tree, both viruses were closely related to viruses from wild birds in central China and East Asian countries; of note, a domestic poultry virus was contained in both sub-branches (Supplementary Figure S1E). For the NS segment, the HH179/H7N7 and CH1228/H7N7 strains were located in separate sub-branches mixed with viruses from domestic poultry and wild birds (Supplementary Figure S1F). These results indicate that the internal genes of both HH179/H7N7 and CH1228/H7N7 are highly similar to viruses circulating in wild birds in the East Asian-Australasian flyway and that the gene pool has been introduced to wild birds and domestic poultry in central China. Although gene flow from the wild birds’ gene pool to domestic poultry is evident both in HA, NA and the internal genes of H7N7 viruses, we could not exclude the possibility that gene flow also occurred from poultry to wild birds.

More phylogenetic details, especially the time scaled evolution, were revealed by the Bayesian phylogenetic reconstruction and demonstrated by maximum clade credibility (MCC) trees. (Supplementary Figure S2A-2H). The PB2, HA and NA segments have a higher evolutionary rate than the other five segments (Table 1). A similar pattern was observed in our previous study of avian H5N6 influenza viruses^11^.

**Table 1:** Evolution rate of eight segments

Segment	Mean (substitution/site/year)	95% HPD interval^a^
PB2	3.0181E-3	[2.6694E-3, 3.384E-3]
PB1	2.0846E-3	[1.7537E-3, 2.4438E-3]
PA	1.6295E-3	[1.44E-3, 1.8162E-3]
HA	3.7418E-3	[2.7253E-3, 4.7226E-3]
NP	2.0786E-3	[1.4761E-3, 2.6873E-3]
NA	4.1986E-3	[3.7492E-3, 4.6703E-3]
MP	1.5698E-3	[1.2418E-3, 1.9081E-3]
NS	1.9612E-3	[1.4596E-3, 2.5017E-3]

^a^HPD: highest posterior density

The MCC phylogenetic trees also indicated the timing of evolution events. The tMRCA of the two H7N7 HA genes existed in September 2012, and its 95% HPD interval was [Apr. 2012–Feb. 2013]. This lineage comprized mixed virus strains isolated from wild birds, ducks, chickens and the environment. The tMRCA of HH179/H7N7 and CH1228/H7N7 NA genes was suggested to have existed in June 1999 [Jul. 1998–May 2000], which suggested that these two NA genes became evolutionarily diversified more than ten years ago. The tMRCA of the HH179/H7N7 and CH1228/H7N7 NA genes along with neighbor strains that originated from fowl emerged in September 2012 [Apr. 2012–Feb. 2013] and July 2013 [Apr. 2013–Oct. 2013], respectively. Like the NA gene, the six internal segments of the two strains became evolutionally separated several years ago, but the tMRCA of related linages existed between 2011 and 2013 (Table 2). Thus, reassortment and emergence of these two H7N7 viruses in wild birds might have occurred between mid 2011 and late 2013.

**Table 2:** Time of most recent common ancestor (tMRCA) for each of the eight segments

Segment	Most recent common ancestor	Posterior Probability	tMRCA	95% HPD
PB2	CH1228 and Vietnam duck H3N2	1.0000	Feb. 2013	[Aug. 2012, Jul. 2013]
	HH179 and Jiangxi chicken H7N7	0.9983	Aug. 2013	[May, 2013, Oct. 2013]
PB1	CH1228 and Mongolia duck strains	0.8029	Jun. 2012	[Jul. 2011, May. 2013]
	HH179 and waterfowl, duck and wild bird strains in China	0.1523	Jul. 2011	[Dec. 2010, Mar. 2012]
PA	CH1228 and Jiangxi duck H10N7	1.0000	Mar. 2012	[Sep. 2011, Sep. 2012]
	HH179 and Jiangxi duck H7N3	1.0000	Aug. 2011	[Nov. 2010, Jun. 2012]
HA	CH1228, HH179 and Jiangxi chicken H7N7	1.0000	Sep. 2012	[Apr. 2012, Feb. 2013]
NP	CH1228 and Jiangxi duck strains	0.9916	Sep. 2012	[Jan. 2012, Mar. 2013]
	HH179 and Jiangxi H10N3	0.1721	Jun. 2012	[Nov. 2011, Dec. 2012]
NA	CH1228 and Jiangxi chicken H7N7	1.0000	Sep. 2012	[Apr. 2012, Feb. 2013]
	HH719 and Jiangxi chicken H7N7	1.0000	Jul. 2013	[Apr. 2013, Dec. 2013]
MP	CH1228 and miscellaneous duck, waterfowl and wild bird strains	0.2621	Nov. 2012	[Mar. 2012, Jun. 2013]
	HH179 and Mongolia duck H3N8	0.1357	Mar. 2013	[Jan. 2012, Sep. 2013]
NS	CH1228 and duck, wild bird strains	0.0813	Nov. 2013	[Jun. 2013, Feb. 2014]
	HH179 and Jiangxi chicken H7N7	1.0000	May. 2013	[Sep. 2012, Nov. 2013]

### Molecular markers of migratory waterfowl H7N7 viruses

The monobasic cleavage site in the migratory waterfowl H7N7 isolates was LPKGR/GL (Supplementary Table 2), which suggests that they are of low pathogenicity to poultry. The migratory waterfowl viruses also encoded a full-length NA protein, with no amino acid deletion in the stalk region of NA-a mutation, which has been associated with viral adaptation to land poultry after introduction from wild aquatic birds^12–14^, further implying the low pathogenicity of these viruses for poultry. Since the residues at positions 591, 627 and 701 of the PB2 are thought to be critical for the mammalian adaptation of AIVs, previous studies have shown that single Q591K, E627K or D701N mutations could increase polymerase activity, viral replication in mammalian cells and the pathogenicity of influenza viruses in the BALB/c mouse model^15–18^. Residues Q591, E627, and D701 in the PB2 protein of wild bird H7N7 viruses suggest that these viruses have not yet adapted to mammalian hosts. Furthermore, no drug resistance-associated mutations (H274Y in NA; L26P, V27A, A30T or S31N in M2^19, 20^) were observed, indicating that both migratory waterfowl H7N7 isolates should still be sensitive to NA and M2 inhibitors.

### Environmental stability of migratory waterfowl H7N7 viruses in water under variable conditions of temperature and pH

**Fig. 3:**
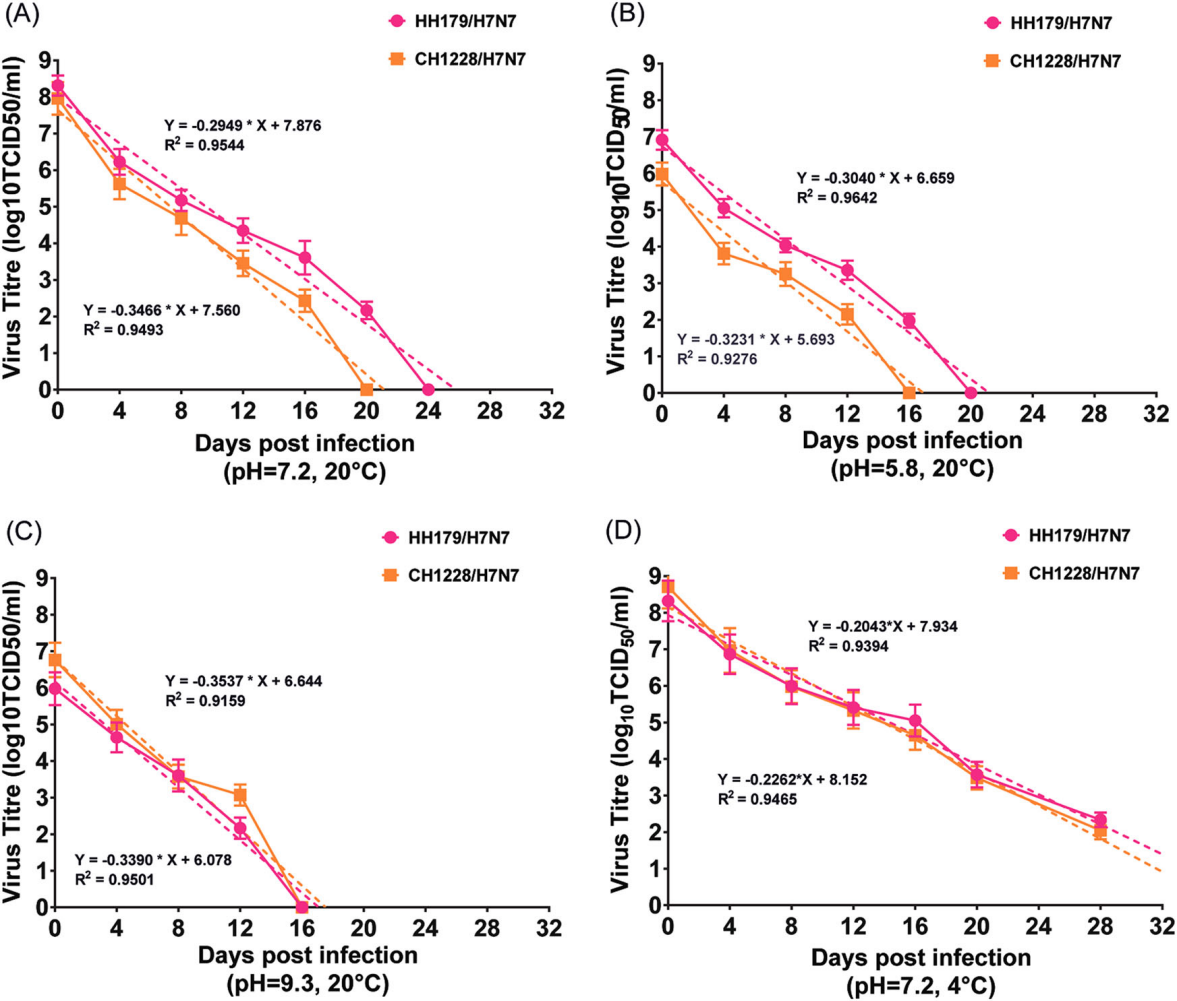
Persistence of migratory waterfowl H7N7 viruses in water under various conditions of temperature and pH. Samples were collected at corresponding days post infection, and viral titers were determined by TCID_50_. Based on the viral titers, regression lines were simulated, and the estimated regression days of both H7N7 viruses were determined. All experiments were performed in triplicate for each water sample, and the titers at individual sampling dates represent the averages of triplicate experiments and the S.D. values

The stability of HH179/H7N7 and CH1228/H7N7 in water under variable conditions of temperature and pH were tested. At 20 °C, HH179/H7N7 and CH1228/H7N7 were the most stable at near-neutral pH (7.2), and the estimated regression days were 26 and 21, respectively (Fig. 3a). The stability of both viruses was reduced under either acidic or basic conditions (Fig. 3b, c). Under the condition of near-neutral pH (7.2), both viruses were more stable at 4 °C than at 20 °C, and the estimated regression days were 39 and 36 days for HH179/H7N7 and CH1228/H7N7, respectively (Fig. 3a, d). Furthermore, we noticed that HH179/H7N7 was slightly more stable in water than CH1228/H7N7 under the various conditions tested (Fig. 3a–c). Our results suggest that wild bird-origin H7N7 viruses can maintain an infectious state for more than two weeks in water.

### Cross-reactive antibody responses between migratory waterfowl H7N7 and human H7N9 isolates

**Fig. 4:**
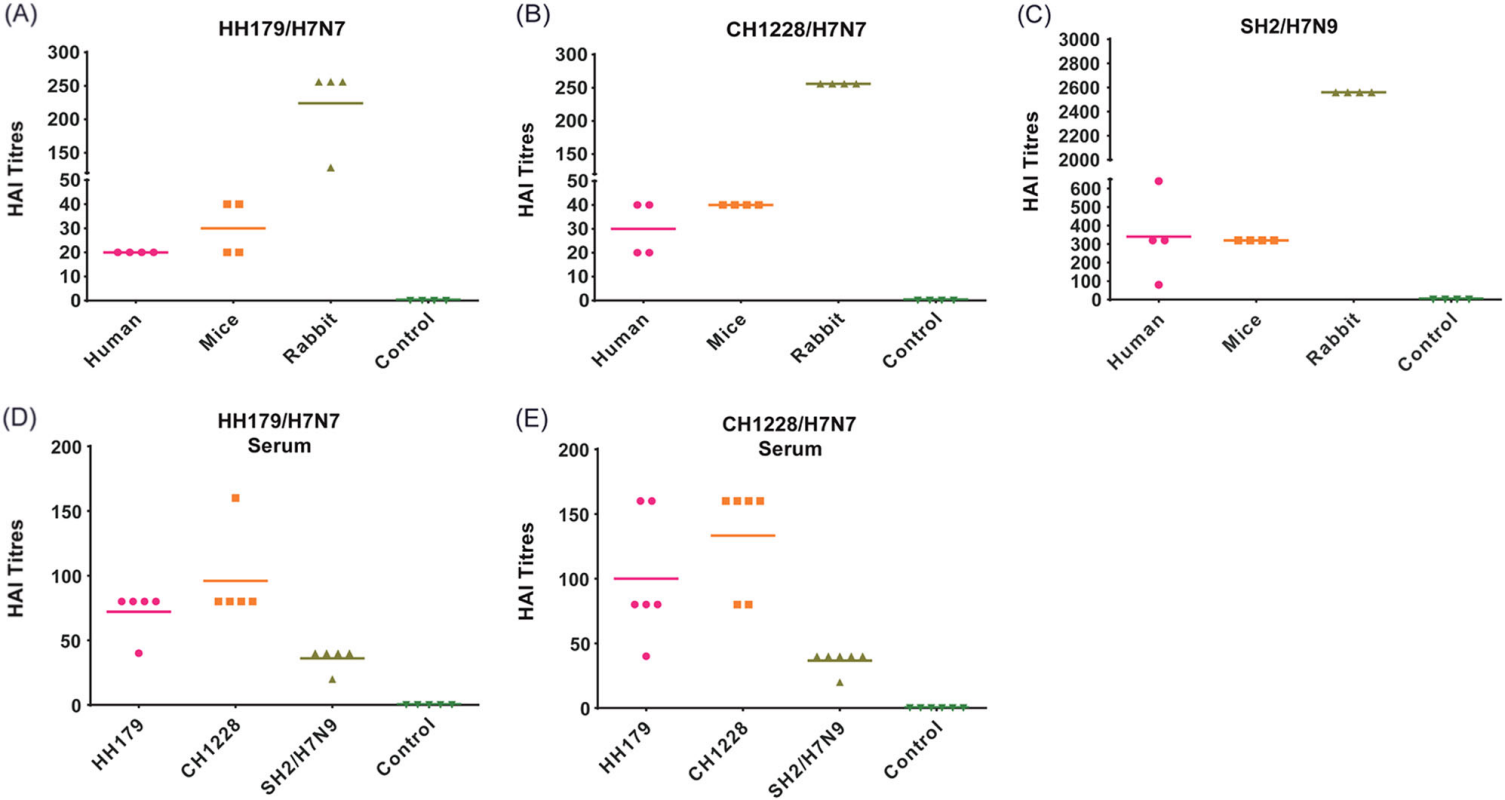
Cross-reactive antibody responses between migratory waterfowl H7N7 and human H7N9 strains. **a**–**c** Anti-serum from human H7N9 patients, H7N9 infected mice and immunized rabbit were tested for HAI titers against heterologous H7N7 and homologous H7N9. **d**, **e** Anti-serum from mice infected with HH179/H7N7 and CH1228/H7N7 were tested for HAI titers against homologous H7N7 and heterologous H7N9 viruses. The control sera were from non-exposed humans, naive mice, non-immunized rabbits (**a**–**c**) and naive mice (**d**,** e**). No HAI activity was detected with control sera, and the data are only shown as a control column

In 2013, avian H7N9 influenza viruses were reported to be infecting people in China, resulting in high mortality. Genetic analysis indicated that the HA gene of the H7N9 virus originated from wild bird H7 viruses. Several previous studies have shown that divergent H7 immunogens induced cross-reactive antibody responses to H7N9 viruses and even provided protection in animal models^21–25^, suggesting that the divergent H7 HA protein shared conserved epitopes. Homologous analysis of the HA protein between HH179/H7N7 and SH2/H7N9 (A/Shanghai/02/2013(H7N9)) showed 95.2% similarity, although the nucleotide sequence identity was only 90.1% (data not shown). Therefore, cross-reactive responses between migratory waterfowl H7N7 and human H7N9 isolates may exist. To clarify this finding, we first tested anti-serum from human H7N9-infected patients, mice recovered from H7N9 infection and H7N9-vaccinated rabbits against HH179/H7N7 and CH1228/H7N7 (Fig. 4a–c). As controls, sera from non-exposed humans, naive mice, and non-immunized rabbits were tested in parallel. The results showed that human anti-serum had a range of 1:80–1:640 HAI titers against SH2/H7N9 virus. The anti-serum of mouse and rabbit origins had high HAI titers against homologous SH2/H7N9 virus, with titers of 1:320 and 1:2560, respectively (Fig. 4c). No HAI activities were detected with control sera (data not shown). Apparently reduced HAI titers against both HH179/H7N7 and CH1228/H7N7 with 2–32, 8–16, and 10–20-fold reductions were observed in each species (humans, mice, and rabbit, respectively) (Fig. 4a, b). Consistently, anti-serum from mice recovered from HH179/H7N7 and CH1228/H7N7 infection displayed high HAI titers against either H7N7 viruses, but it showed low titers against the human isolate SH2/H7N9 (Fig. 4d, e). These results indicate that H7N7 viruses from wild birds have modest but limited cross-reactive antibody responses with the divergent 2013 human H7N9 virus.

### Migratory waterfowl H7N7 viruses replicated well in mammalian cells but inefficiently in mice

**Fig. 5:**
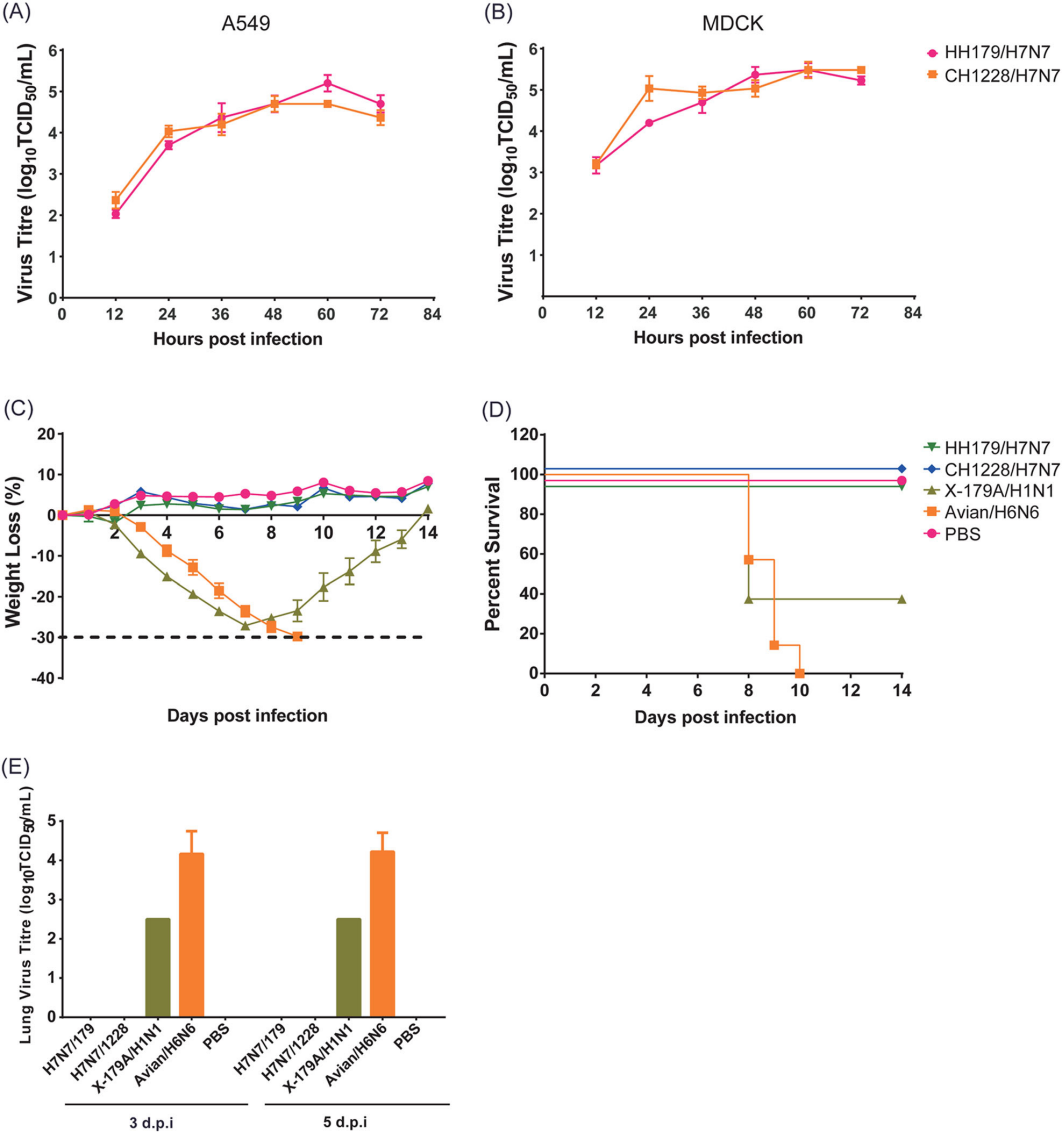
In vitro and in vivo infection of H7N7 viruses. Multiple-cycle growth curves of migratory waterfowl H7N7 viruses in A549 (**a**) and MDCK (**b**) cells. Pathogenicity of the H7N7 viruses was analyzed in BALB/c mice. As controls, mouse-adapted poultry strain A/duck/Hubei/5/2010 (H6N6) and 2009 pandemic vaccine strain NYMC X-179A (A/reassortant/NYMC X-179A (California/07/2009 × NYMC X-157) (H1N1)) were infected in parallel. HH179/H7N7 and CH1228/H7N7 were inoculated into the mice of each group by the intranasal route at a dose of 10^6^ EID_50_. Mice were infected with 5 MLD_50_ H6N6 and 1 MLD_50_ NYMC X-179A. Mice in the control group were mock infected with phosphate-buffered saline (PBS). **c** Body weights were monitored daily for a 14-day observation period, and weight changes are expressed as a percentage of the initial value (measured on the day of challenge). **d** Kaplan–Meier survival curve. **e** Lung viral titers were determined at 3 and 5 dpi by TCID_50_ on individual lung homogenates from infected mice

To evaluate the replication capability of migratory waterfowl H7N7 viruses, the kinetics of infectivity were determined by multiple-cycle growth curves in MDCK and A549 cells. The results showed that both H7N7 viruses replicated efficiently in MDCK and A549 cells and that they replicated to slightly higher titers in MDCK than in A549 cells (Fig. 5a, b). Notably, both H7N7 viruses replicated to significantly higher titers in MDCK cells at 12 h post infection than in A549 cells.

The pathogenicity of both viruses in an animal model was further determined in BALB/c mice. As controls, mouse-adapted poultry strain A/duck/Hubei/5/2010 (H6N6)^26^ and 2009 pandemic vaccine strain NYMC X-179A (A/reassortant/NYMC X-179A (California/07/2009 × NYMC X-157) (H1N1))^27^ were infected in parallel. For H7N7 strain infection, each group of mice was inoculated intranasally with a viral dose of 10^6^ EID_50_. Mice were infected with 5MLD_50_ H6N6 and 1MLD_50_ NYMC X-179A. After infection, virus replication in organs, weight loss and mortality in mice were examined. Mice infected with mouse-adapted H6N6 and NYMC X-179A experienced significant weight loss (>25%) and displayed a low survival rate (Fig. 5c, d). In contrast, mice infected with HH179/H7N7 and CH1228/H7N7 showed no clinical symptoms after infection (Fig. 5c). All mice in the H7N7 infection group survived during the 2-week observation period (Fig. 5d). The titers of virus replication in lungs, spleen, kidney and brain were examined at 3 and 5 days post-infection, and no viruses were detected in mice infected with H7N7 strains (data not shown). In line with severe clinical symptoms, high lung virus titers in mice infected with H6N6 and NYMC X-179A were observed at 3 and 5 dpi (Fig. 5e). These data suggest that both of the wild bird-origin H7N7 viruses in this study are avirulent in mice and that viral replication is inefficient in this host.

## Discussion

Previous investigations provided evidence of the circulation of H7N7 viruses in wild aquatic birds in East Asian countries located on the East Asia-Australasian flyway, although the frequency of isolation differed by location, time and host. In South Korea, the results of AIV surveillance in wild birds indicated that H7N7 viruses were frequently isolated from feces or oropharyngeal swab samples between 2006 and 2014^28–31^. In other countries, 5-year surveillance of migratory waterfowl indicated that H7N7 viruses have been isolated in Japan both in 2010 and 2011 and in Mongolia in 2012^32^. In China, which is also located on the flyway, only a single paper reported the isolation of an H7N7 virus from a wild goose in 2012 in East Dongting Lake^33^, indicating that the frequency of isolation of the H7N7 virus in this area was rare. Our results further confirm the occurrence of ongoing H7N7 circulation. In this study, active surveillance of AIVs in migratory birds was undertaken during December 2013–March 2014, and two H7N7 strains (HH179/H7N7 and CH1228/H7N7) were recovered from the fresh droppings of migratory birds in different wetlands. However, since then, no H7N7 viruses were isolated from wild migratory birds in the following 3-year surveillance (data not shown).

The results of genetic and phylogenetic analysis indicated that all eight segments of the two H7N7 viruses originated from the Eurasian wild bird low pathogenic avian influenza (LPAI) gene pool; then, they diverged from each other, except for the HA gene. However, an overall view of the diversity of the H7N7 virus circulating in the East Asia-Australasian flyway could not be extrapolated since the sample sizes, sampling sites and isolation frequency were restricted. In addition to genetic diversity, we noticed that most genes from both of the H7N7 viruses were highly similar to those of domestic poultry viruses, indicating that genes from the wild bird virus gene pool had been introduced into domestic poultry. The introduction of the wild bird LPAI gene pool into domestic poultry has been proven to contribute to the emergence of novel epidemic strains. Analysis of the origin and source of the H7N9 virus in China in 2013 indicated that the ancestral H7 viruses from wild birds were introduced into domestic ducks in China and led to the 2013 H7N9 outbreak lineage viruses^34^. In late 2013 and early 2014 in Jiangxi, China, the first case of human infections with H10N8 virus was identified^35^. A long-term influenza surveillance of migratory birds and poultry in Southern China showed that H10 influenza viruses have been introduced from migratory to domestic ducks over several winter seasons at Poyang Lake^36^. Although the introduction event observed in this study is evident, whether these introduced genes contribute to the generation of a novel emerging virus strain requires further long-term surveillance in both migratory birds and domestic poultry.

Geographical separation of the host species has shaped H7 AIVs into independently evolving Eurasian and American lineages (Fig. 2). Among each lineage, the H7 AIVs circulating in migratory birds have similar antigenic epitopes and efficiently cross-react with each other. In North America, the antigenic and genetic characterization of 85 H7 AIV isolates from migratory waterfowl (1976–2010) indicated that genetic diversity has not led to significant antigenic diversity for H7 viruses^37^. In Asia, the antigenic analysis results indicated that the antigenicity of viruses in wild water birds is highly stable despite their nucleotide sequence diversity^28, 29, 31, 32^. Our results also showed limited antigenic diversity between the two H7N7 viruses, which was consistent with the genetic and phylogenetic results. In this study, we also tested cross-reactive antibody responses between wild bird H7N7 viruses and the 2013 H7N9 virus, and the results showed that the H7N7 viruses had moderate cross-reactive antibody responses with the divergent 2013 H7N9 virus. Previous studies have shown that vaccinations with divergent heterologous H7 immunogens from both Eurasian and American lineages could raise cross-reactive hemagglutination-inhibiting antibodies against H7N9 viruses and even protect mice from lethal viral infections^38–42^. Similar cross-reactivity was observed in the sera of human subjects from a clinical trial with a divergent H7 vaccine^24, 25, 43^. The basis for cross-reactivity may lie in the conserved antigenic sites on the HA protein within the divergent H7 subtype^39^.

AIVs remain infectious for weeks in low-temperature waters, and water bodies have been considered to be an important route for the spread of AIV^44–47^, yet few reports exist on the persistence of infectivity of wild bird-origin H7N7 viruses in water bodies. Improved knowledge of the influence of environmental factors on the persistence of AIV would be valuable for risk assessments. In this study, the stability of HH179/H7N7 and CH1228/H7N7 in water under variable conditions of temperature and pH were tested. Our results suggest that wild bird-origin H7N7 viruses could maintain their infectivity for more than two weeks in water, representing a potentially important route for the dissemination of H7N7 viruses, especially during the autumn and winter seasons. One limitation of our study is that the stability of H7N7 viruses have not been compared with that of other influenza strains. To date, many studies have evaluated the environmental factors (temperature, salinity, and pH) that influence the stability of different AIVs under variable conditions. Overall, the persistence of AIVs was negatively associated with the temperature and acidity of the environment, and it has been suggested that AIVs remains infective for the longest time between a pH of 7.2 and 8.4 at low temperature^46, 48, 49^. In line with previous studies on viral persistence, our study also showed that both HH179/H7N7 and CH1228/H7N7 were more stable under the condition of near-neutral pH (7.2) and cold temperature (4 °C) than under other conditions. In addition, Poulson et al.^46^. evaluated the ability of eight swine (H1N1 and H3N2) and six human (H1N1 and H3N2) strains to remain infective under various pH, temperature, and salinity conditions. In addition, the results showed that no significant differences in persistence were observed between pandemic and nonpandemic strains. A similar study also observed no significant differences in persistence between avian origin H5N1 and H9N2 strains^47^. Based on the fact that no significant phenotypic diversity in response to temperature and pH has been suggested, we speculated that differences will be observed between wild bird-origin H7N7 strains and other influenza viruses.

The H7N7 viruses were found to replicate well in cells but inefficiently in mice since the virus could not be recovered from lungs or other organs. These data suggest that both of the wild bird-origin H7N7 viruses in this study are avirulent in mice, which is consistent with the absence of mammalian adaptation-associated Q591K, E627K, or D701N mutations in the PB2 protein of H7N7 viruses. In summary, two genetically diverse H7N7 viruses were recovered from the fresh droppings of different migratory birds. Of note, some genes of the H7N7 viruses originating in wild birds have been introduced into domestic poultry. Although the isolation frequency of H7N7 viruses in the East Asian-Australasian flyway in this study is low, long-term surveillance of this subtype in migratory birds should be performed to facilitate forewarning of the circulation of H7N7 viruses.

## Materials and methods

### Ethics statement

All studies and procedures involving animals were conducted in accordance with the animal welfare guidelines of the World Organization for Animal Health. Experimental protocols were approved by the Animal Welfare and Ethical Review Committees of Wuhan Institute of Virology, Chinese Academy of Sciences.

### Sampling

Chenhu wetlands (N 30°15′10″–30°24′44″, E 113°44′07″–113°55′39″) and Honghu wetlands (N 29°39′–30°12′, E 113°7′–114°05′) are located in Hubei Province and close to the Yangtze River (Fig. 1). These wetlands are separated from each other by approximately 60 kilometers and located within the East Asian-Australasian flyway (Fig. 1). In the winter and spring, migratory waterfowl aggregate at both wetlands, making it an ideal sampling site for AIV surveillance. To observe AIVs in migratory birds, fresh and well-separated droppings of wild birds were sampled using sterile swabs in these wetlands from December 2013 to March 2014. Each sample was placed in a vial containing 2 mL of viral transport medium, stored at 2–8 °C, and shipped to the laboratory within 12 h for further analysis.

### Virus isolation and sequencing

The specimens were inoculated into the allantoic cavities of 10-day-old specific-pathogen-free embryonated eggs (Beijing Merial Ltd, Beijing, China). After incubation at 37 °C for 72 h, the allantoic fluid of the inoculated eggs was collected and tested for the presence of hemagglutinin activity. AIVs were confirmed in the positive samples by reverse transcription PCR. The hosts of the AIV-positive samples were identified by mitochondrial analysis, as described previously^50^.

All gene segments were amplified with *Ex Taq* DNA polymerase (Takara, Beijing, China) with segment-specific primers, as described previously^51^. The PCR products were purified and sequenced with an ABI 3730 DNA Analyzer (Applied Biosystems). The data were edited and aligned with DNAMAN (version 7.0) and BioEdit (version 7.0.5.2). The whole-genome sequences of two strains of H7N7 influenza isolates were determined and deposited in GISAID (accession numbers: EPI740603- EPI740610 and EPI740659- EPI740666).

### Sequence analysis

Eight data sets were used for the phylogenetic inferences of all segments. The segment 4 and 6 datasets included all available H7 and N7 segments from GenBank and GISAID databases. The dataset of each internal segment was derived from the top 200 BLASTN^52^ hits against a joint database composed of influenza A virus sequences from GenBank and GISAID. In all datasets, identical sequences and sequences without definite collection dates were removed to accelerate phylogenetic inference and improve the precision of the analysis.

The alignment of each dataset was generated with Clustal Omega^53^ (version 1.2.1) with 1000 iterations, and codon alignments were applied to all datasets. Maximum-likelihood phylogenetic trees were inferred with the software RAxML (version 8.2.6) under the GAMMAGTR model with 1000 bootstraps^54^. The time of the most recent common ancestor (tMRCA) for all segments was estimated with BEAST^55^ (version 2.3.2) with the HKY85 plus Gamma nucleotide substitution model and a relaxed clock.

### Antibody analysis

Anti-serum from four human H7N9 infection cases^56, 57^ was provided by Nanjing Drum Tower Hospital. Written informed consent was obtained from all patients involved in this study. Sera from mice were collected 3 weeks after each mouse was infected with an 10^5^ 50% egg infectious dose (EID_50_) H7N9 (A/Shanghai/02/2013) or H7N7 (HH179/H7N7 and CH1228/H7N7) in 20 µl of phosphate-buffered saline (PBS). Rabbit anti-sera were raised by sequentially immunizing rabbits with three doses of whole inactivated SH2/H7N9 (200 µg/dose) at 3-week intervals and were collected at 3 weeks post last booster. The hemagglutination inhibition (HAI) assay was performed with 0.5% chicken red blood cells, as described previously^58^. The HAI titers were determined as the reciprocal of the highest serum dilution that completely inhibited hemagglutination.

### Virus persistence tests

HH179/H7N7 and CH1228/H7N7 were propagated in 10-day-old specific pathogen free (SPF) embryonated chicken eggs, and allantoic fluid was harvested 72 h later. The stock titers of HH179/H7N7 and CH1228/H7N7 were determined in Madin–Darby Canine Kidney (MDCK) cells and were 10^9.20^ TCID_50_/mL and 10^8.4^ TCID_50_/mL, respectively. Distilled water was buffered with 10 mM HEPES (Sigma, St. Louis, MO). For pH trials, water pH was adjusted to 5.8, 7.2, and 9.3 with the addition of 1 N HCl or NaOH. Each pH treatment was measured at the start of the study and confirmed at the completion of each trial. HH179/H7N7 and CH1228/H7N7 viruses were diluted 1:100 in the treated water samples in different pH conditions. The inoculated water samples were then divided into 1.0-mL aliquots in 2.0-mL polystyrene tubes and placed in incubators (three aliquots in each sampling point). The viral inoculated water was sampled at the time of viral inoculation (0 days post infection) and every 4 days post inoculation. The virus infectivity in water samples in each time point was determined by microtiter end-point titration, and the results were expressed as 50% tissue culture infectious dose (TCID_50_)/mL, as described previously^46^. The calculation of TCID_50_ was based on the Reed–Muench method. Linear regression was used to determine a 90% reduction time (Rt) for each virus-treatment combination; Rt values correspond to the time required for a decrease in the viral titer by 1 log_10_TCID_50_/mL, as described previously^46^. The regression equations are shown in Fig. 3. The minimum detectable limit for this procedure is 10^1.6^ TCID_50_/mL.

### In vitro and in vivo infection

Multiple-cycle growth curves were used to analyze the replication properties of the H7N7 viruses. Madin–Darby Canine Kidney (MDCK) or A549 cell monolayers were inoculated with diluted virus at a multiplicity of infection (MOI) of 0.01. After incubation at 37 °C for 1 h, virus suspensions were removed, and 3 mL of modified Eagle’s medium containing 1.0 μg/mL trypsin was added. Culture media were collected every 12 h for 72 h after infection. The viral titers of each sample were determined by TCID_50_ in MDCK cells and calculated by the Reed–Muench method. Each experiment was performed in triplicate.

The animal experiment was performed, as previously described^59^. Briefly, twelve 6-week-old SPF BALB/c mice were infected intranasally under anesthesia with 20 μl of each virus. On days 3 and 5, three of the inoculated mice were sacrificed for virus titration in the lung, kidney, spleen and brain. Tissue samples were homogenized in 1 mL of cold PBS and centrifuged at 16,000 × *g* for 10 min before the homogenates were titrated for virus infectivity in MDCK cells with initial dilutions of 1:10. The remaining six inoculated mice were monitored daily for weight loss and mortality.

## Electronic supplementary material

Supplementary Figure S1

Supplementary Figure S2

Supplementary Table S1

Supplementary Table S2
