# Supplementary material for: Two genetically diverse H7N7 avian influenza viruses isolated from migratory birds in central China
Source: Emerg Microbes Infect. 2018 Apr 11;7:62. doi: 10.1038/s41426-018-0064-7 (PMC5893581; doi:10.1038/s41426-018-0064-7)

**Supplementary Figure S1**

Maximum likelihood phylogenetic trees of eight segments, inferred by RAxML. Virus strain names in red were virus strains studied in this research, blue were from poultry, and black were wild-bird and environmental samples. (A) PB2; (B) PB1; (C) PA; (D) NP; (E) M; (F) NS.


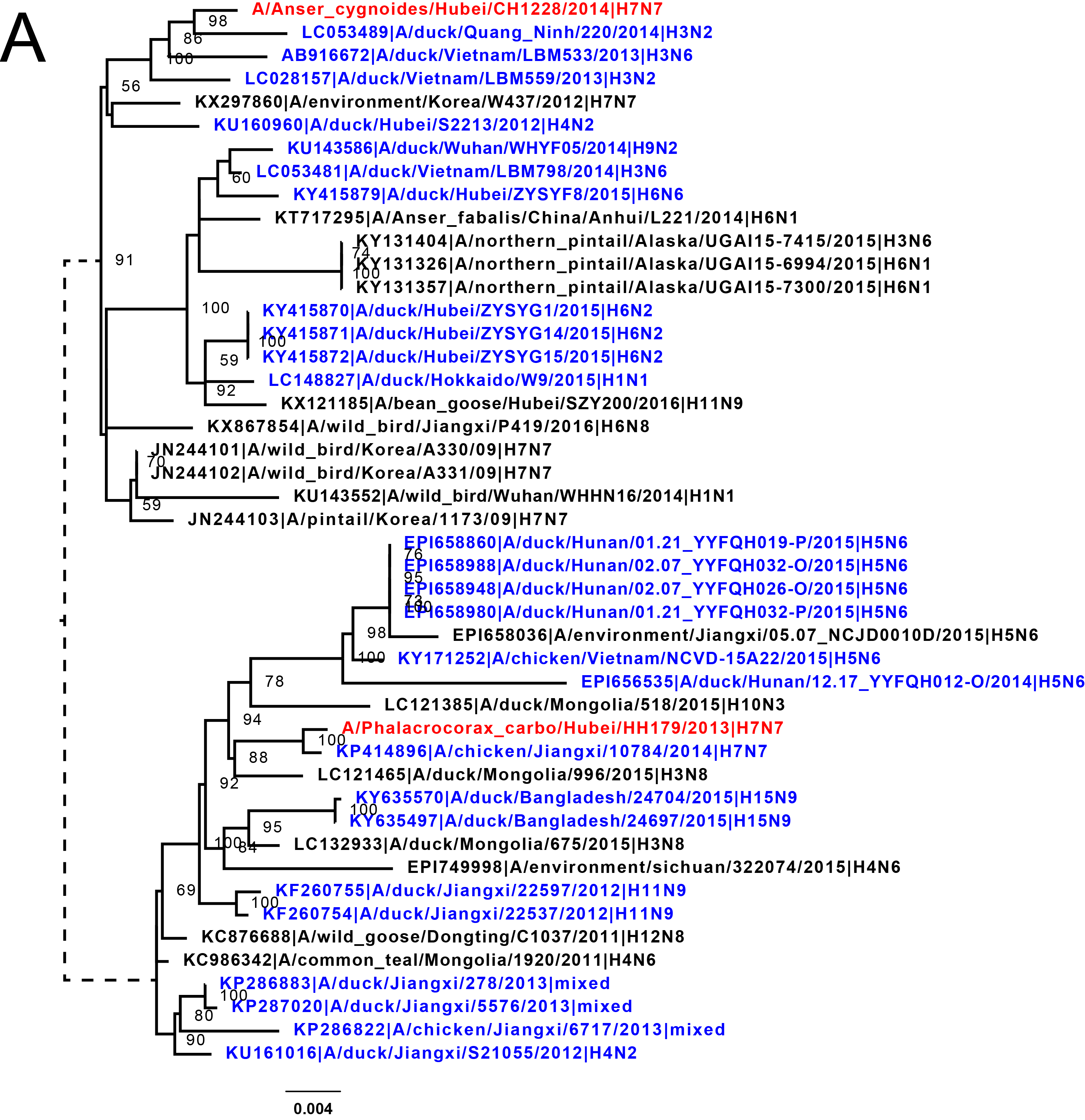


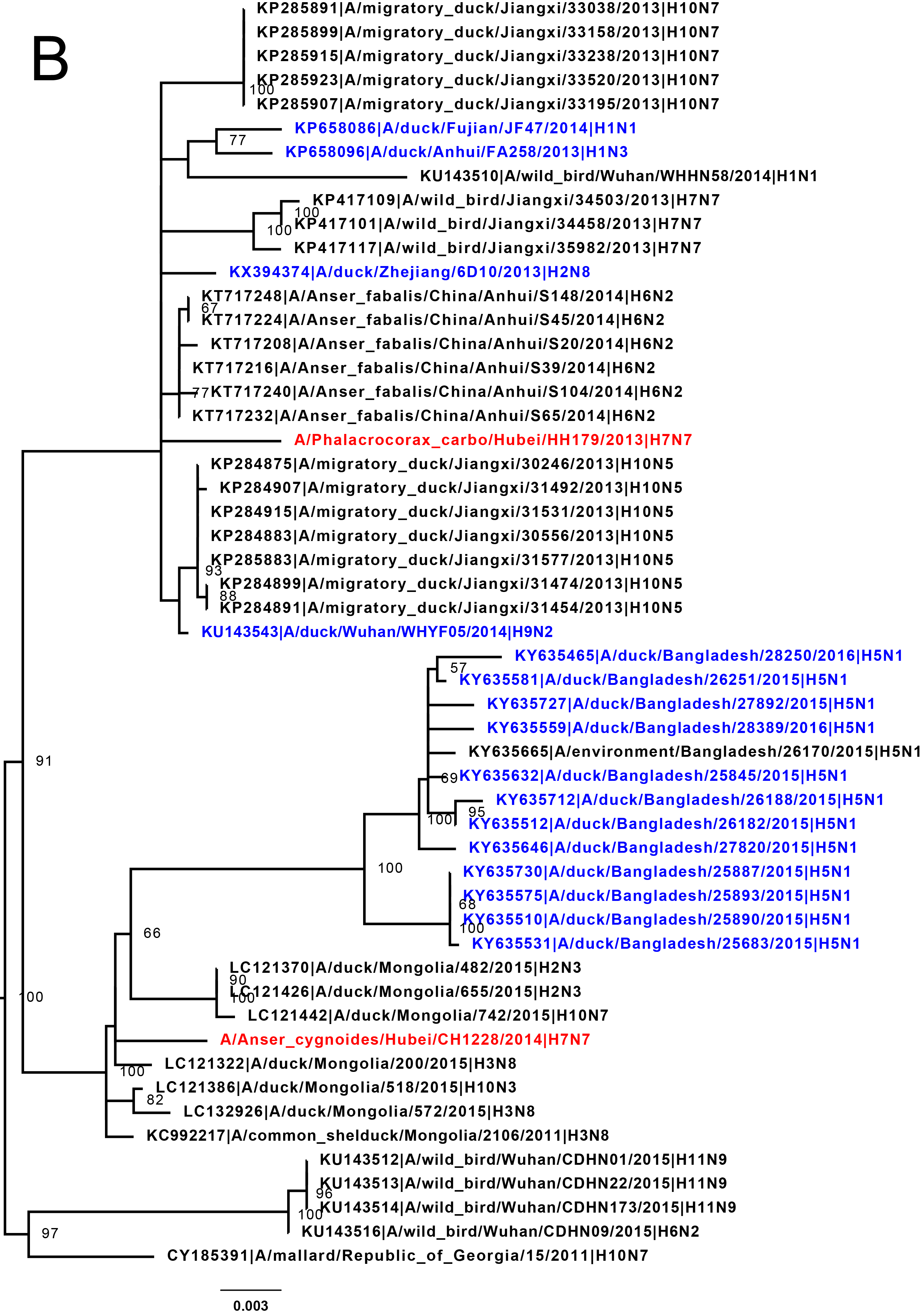


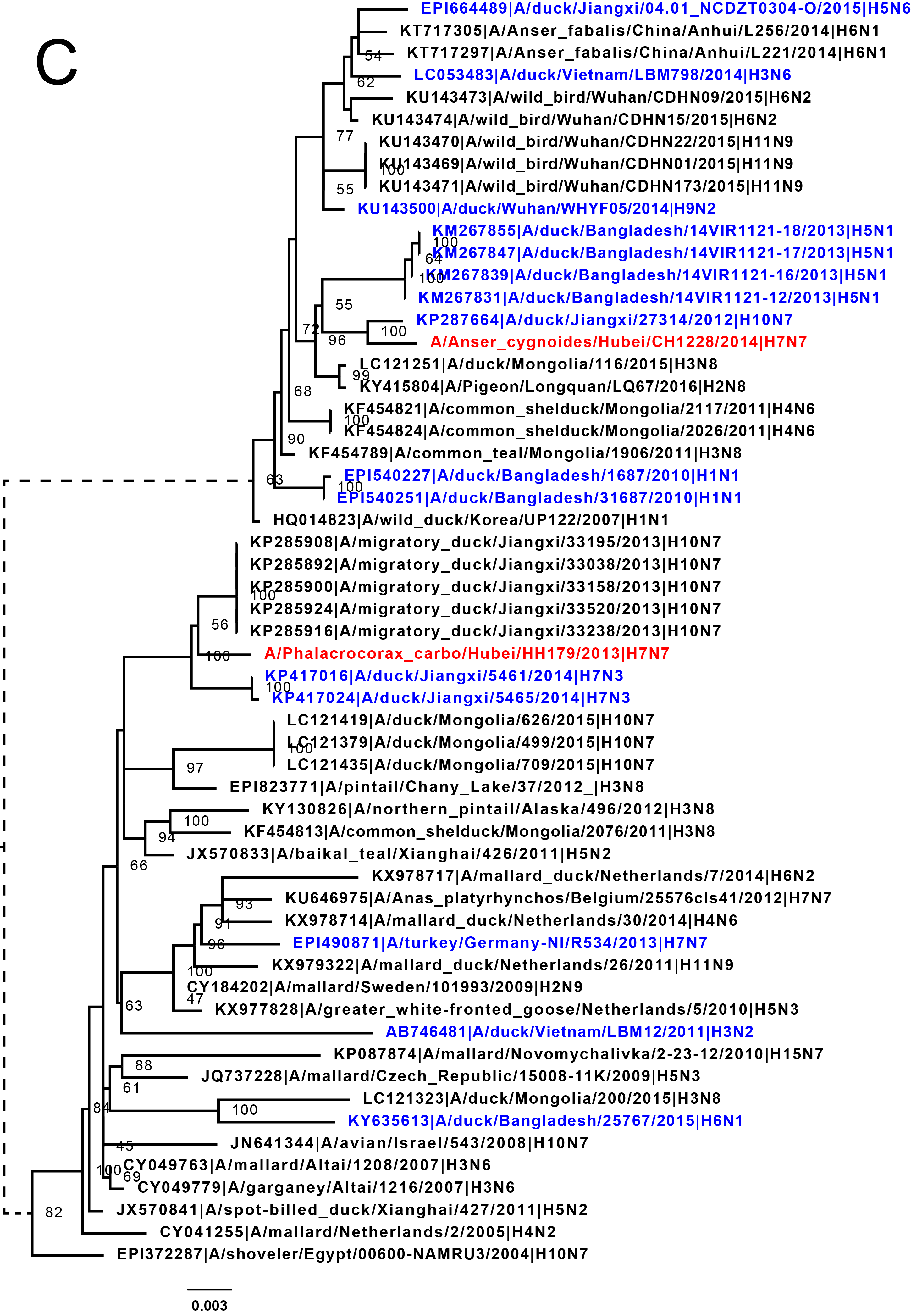


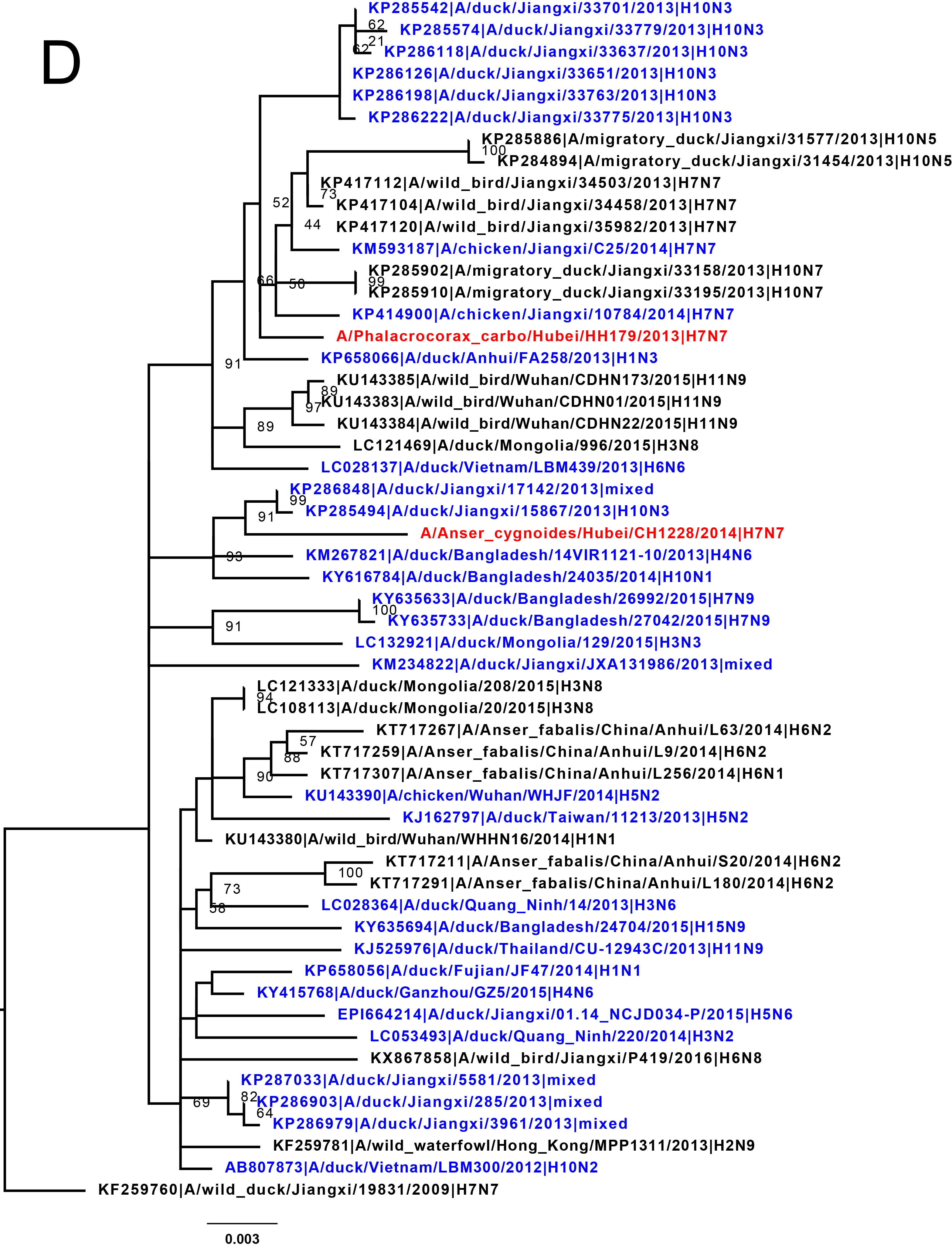


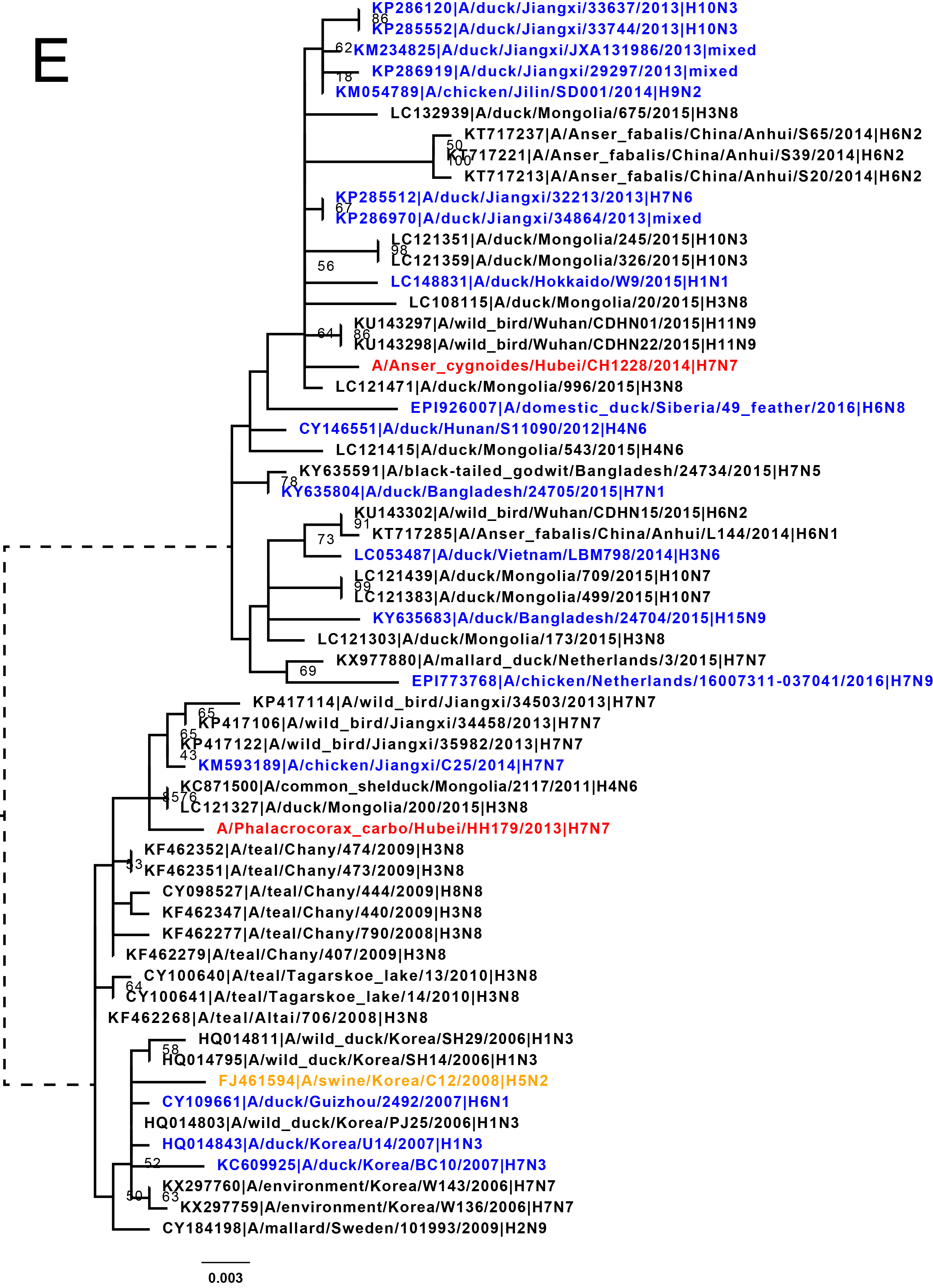


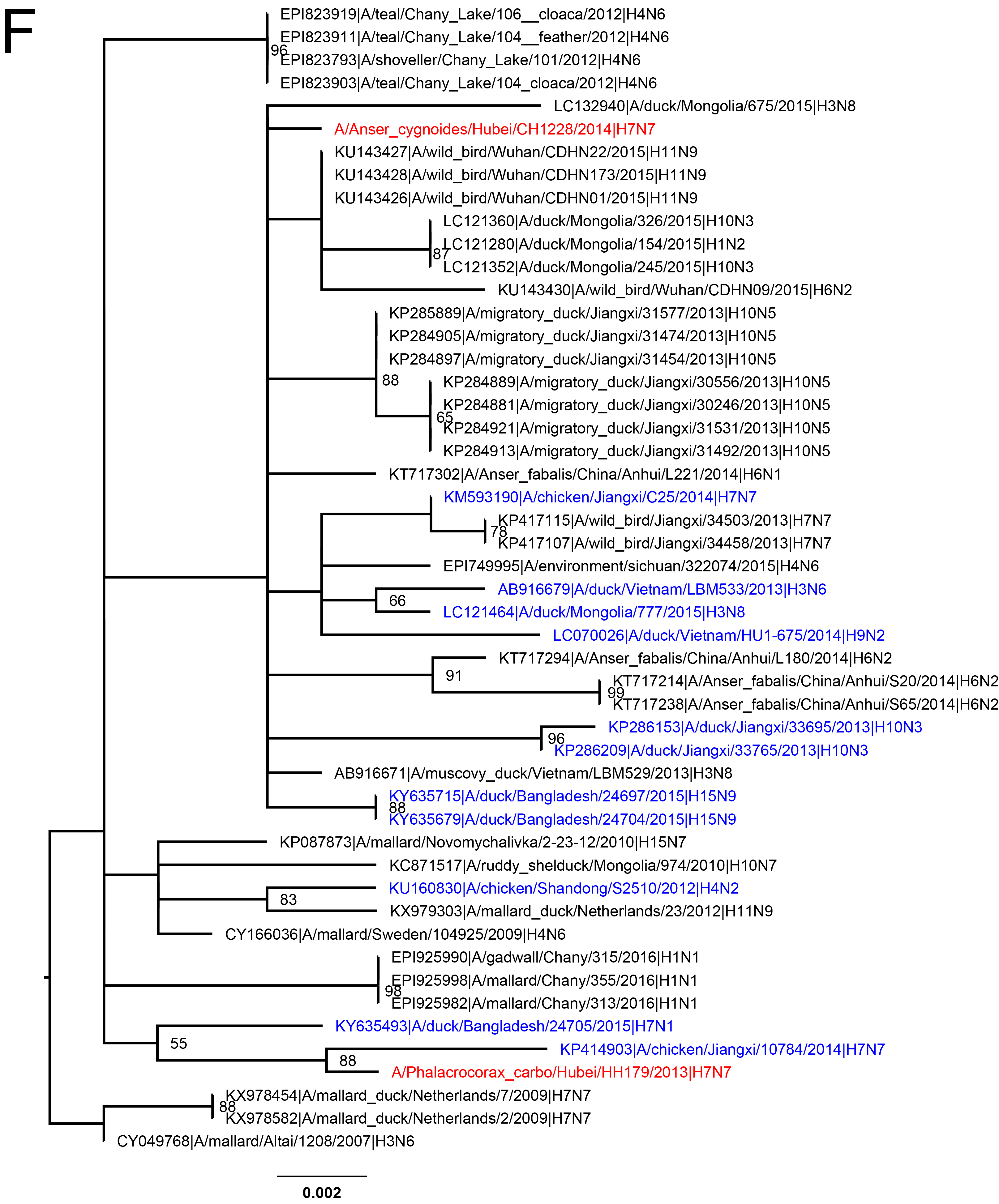

Supplement: Supplementary file 1 — Supplementary Figure S1 [file 41426_2018_64_MOESM1_ESM.doc]
