# Supplementary material for: Two genetically diverse H7N7 avian influenza viruses isolated from migratory birds in central China
Source: Emerg Microbes Infect. 2018 Apr 11;7:62. doi: 10.1038/s41426-018-0064-7 (PMC5893581; doi:10.1038/s41426-018-0064-7)

**Supplementary Figure S2**

Maximum clade credibility trees of eight segments, inferred by BEAST. Virus strain names in red indicated virus strains studied in this research. Node bar indicated height 95% HPD. (A) PB2; (B) PB1; (C) PA; (D) HA; (E) NP; (F) NA; (G) M; (H) NS.


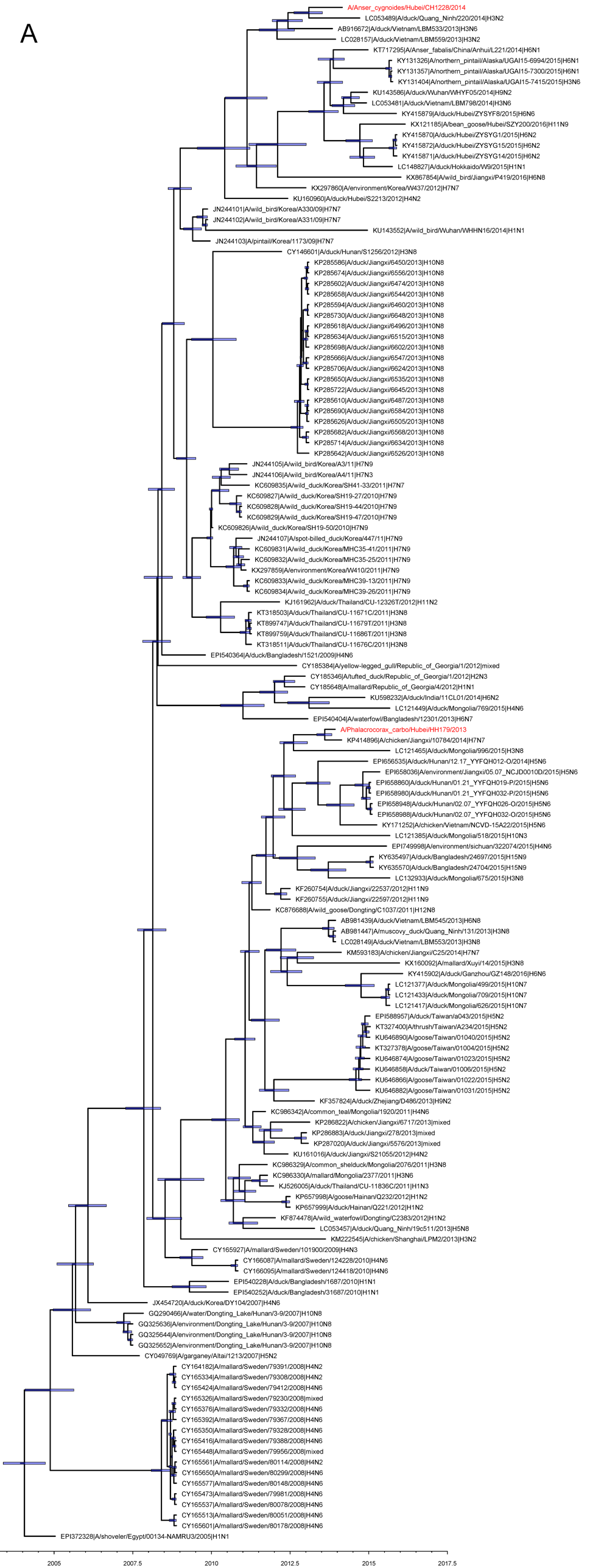


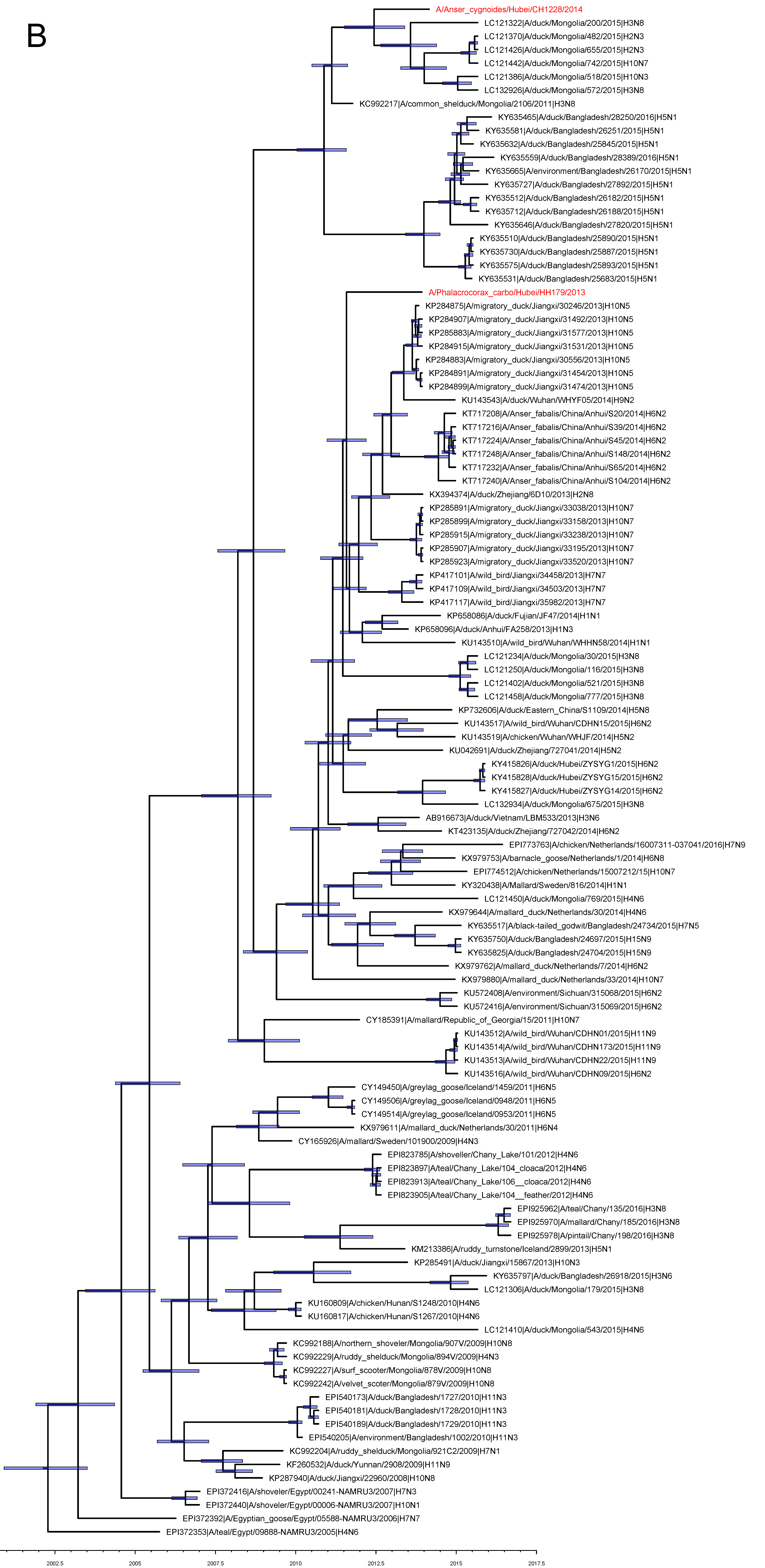





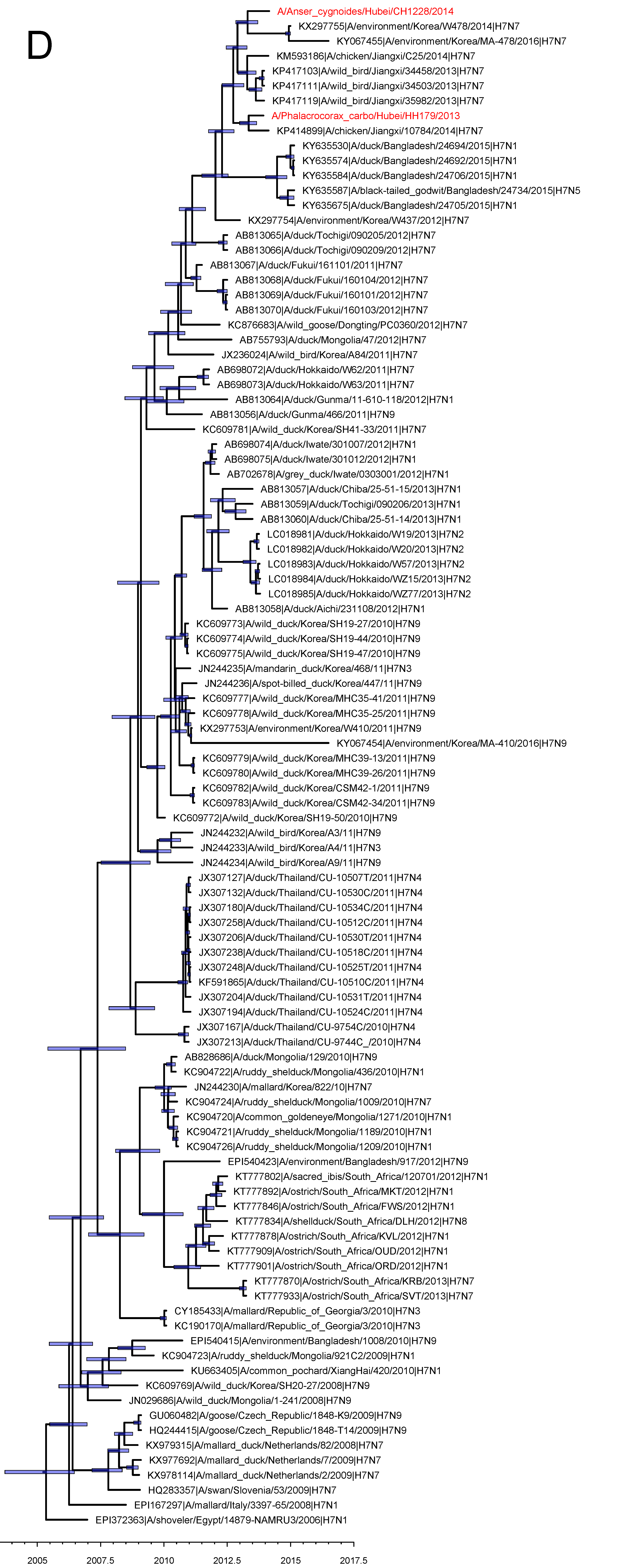


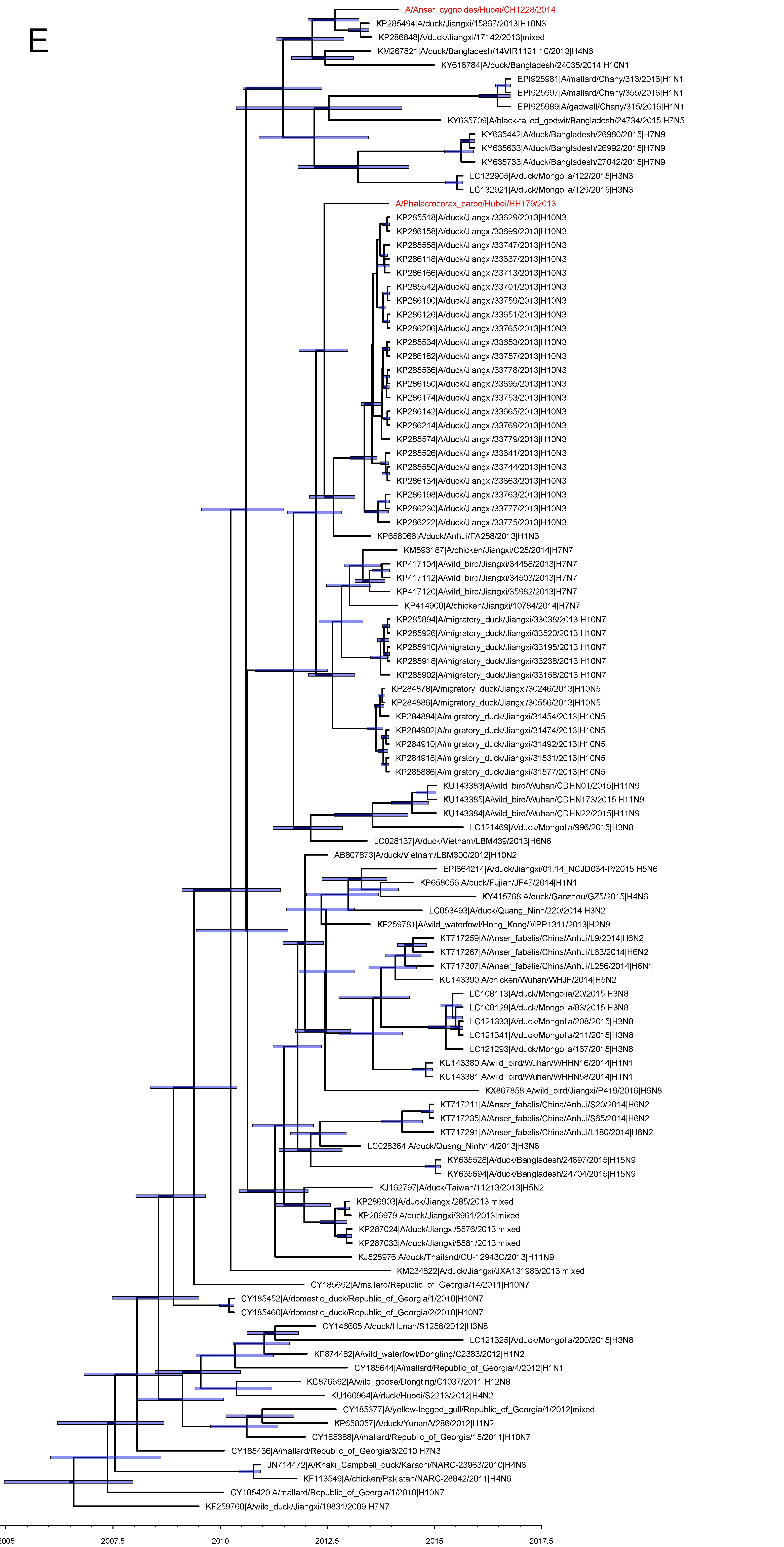








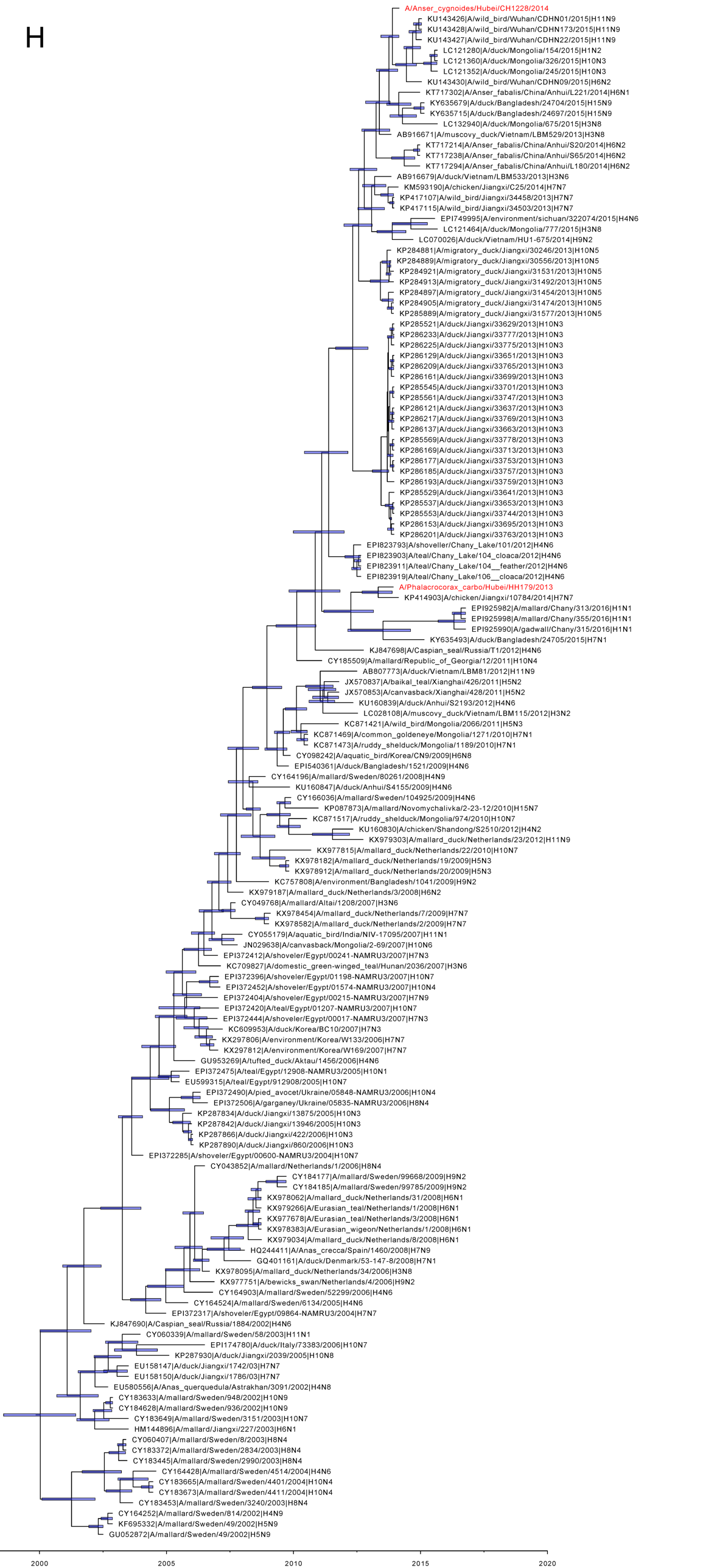

Supplement: Supplementary file 2 — Supplementary Figure S2 [file 41426_2018_64_MOESM2_ESM.doc]
