# Supplementary material for: Two genetically diverse H7N7 avian influenza viruses isolated from migratory birds in central China
Source: Emerg Microbes Infect. 2018 Apr 11;7:62. doi: 10.1038/s41426-018-0064-7 (PMC5893581; doi:10.1038/s41426-018-0064-7)
Supplement: Supplementary file 3 — Supplementary Table S1 [file 41426_2018_64_MOESM3_ESM.doc]

Supplementary Table S1 Genetic identity (%) of the migratory birds H7N7 viruses isolated from Hubei, China

| Strain | Segment | Percent identity of nucleotide (%) | | | |
| --- | --- | --- | --- | --- | --- |
|  |  | HH179/H7N7 | | CH1228/H7N7 | Human/H7N7 ^a^ |
| HH179/H7N7 ^b^ | HA |  | - | 98.6 | 91.7-91.8 |
|  | NA |  | - | 90.6 | 91.5-94.4 |
|  | PB2 |  | - | 97.2 | 93.4-94.6 |
|  | PB1 |  | - | 98.1 | 94.9-95.9 |
|  | PA |  | - | 96.2 | 93.7-96.3 |
|  | NP |  | - | 98.3 | 94.6-95.5 |
|  | M |  | - | 97.3 | 95.3-97.8 |
|  | NS |  | - | 98.9 | 97.7-97.7 |
| CH1228/H7N7 | HA |  | 98.6 | - | 91.9-92.0 |
|  | NA |  | 90.6 | - | 92.3-94.2 |
|  | PB2 |  | 97.2 | - | 93.6-94.6 |
|  | PB1 |  | 98.1 | - | 95.3-96.3 |
|  | PA |  | 96.2 | - | 93.7-95.4 |
|  | NP |  | 98.3 | - | 94.2-95.2 |
|  | M |  | 97.3 | - | 95.8-97.8 |
|  | NS |  | 98.9 | - | 97.8-98.0 |

^a^ The two strain of human infection which are A/Netherlands/219/2003 (H7N7) and A/Italy/3/2013 (H7N7).

^b^ HH179/H7N7 and CH1228/H7N7 referred to strain of A/Phalacrocorax carbo/Hubei/HH179/2013 (H7N7) and A/Anser cygnoides/Hubei/CH1228/2014 (H7N7) isolated in this study, respectively.
