# Supplementary material for: Two genetically diverse H7N7 avian influenza viruses isolated from migratory birds in central China
Source: Emerg Microbes Infect. 2018 Apr 11;7:62. doi: 10.1038/s41426-018-0064-7 (PMC5893581; doi:10.1038/s41426-018-0064-7)
Supplement: Supplementary file 4 — Supplementary Table S2 [file 41426_2018_64_MOESM4_ESM.doc]

Supplementary Table S2 Molecular characterizations of migratory waterfowl H7N7 viruses

| Viruses (H7N7) ^†^ | Collection  date | HA (H3 numbering) | | | | | |  | PB2 | | | | NA | | |  | | M2 | | | | |  | |
| --- | --- | --- | --- | --- | --- | --- | --- | --- | --- | --- | --- | --- | --- | --- | --- | --- | --- | --- | --- | --- | --- | --- | --- | --- |
|  |  | Connecting  peptide |  | 224 | 226 | 228 | 318 |  | | 591 | 627 | 701 |  | 274* | Stalk deletion | |  | | 26 | 27 | 30 | 31 | |  |
| HH179 | 2013-12-05 | LPKGR/GL |  | N | Q | G | T |  | | Q | E | D |  | H | No | |  | | L | V | A | S | |  |
| CH1228 | 2014-2-26 | LPKGR/GL |  | N | Q | G | T |  | | Q | E | D |  | H | No | |  | | L | V | A | S | |  |

*N2 numbering.

^†^HH179, A/Phalacrocorax carbo/Hubei/HH179/2013(H7N7); CH1228, A/Anser cygnoides/Hubei/CH1228/2014(H7N7).
